# Supplementary material for: Captive-bred Atlantic salmon released into the wild have fewer offspring than wild-bred fish and decrease population productivity
Source: Proc Biol Sci. 2020 Oct 21;287(1937):20201671. doi: 10.1098/rspb.2020.1671 (PMC7661298; doi:10.1098/rspb.2020.1671)
Supplement: Supplementary material [file rspb20201671supp1.docx]

**Supplementary material**

Table S1: Studies of salmonine fishes that have examined differences in fitness or components of fitness between wild- and captive-bred conspecifics. ‘Local’ and ‘nonlocal’ stock refers to whether the captive-bred fish used in a study were derived from brood collected locally or from brood collected from a different system i.e. a neighbouring catchment. ‘Y’ = Yes, ‘N’ = No. Details from this study highlighted in bold.

| Publication | Species | Local stock | Non-local stock | Parentage assignment | Cohort number | Molecular markers | Fitness measure(s) | Fitness difference |  |
| --- | --- | --- | --- | --- | --- | --- | --- | --- | --- |
|  |  |  |  |  |  |  |  |  |  |
| Fleming et al 1997 | *Salmo salar* | Y | N | N (tagging) | NA | NA | Male attendance at redds, female redd construction | Y |  |
| McGinnity et al 2003 | *Salmo salar* | Y | N | Y | 1 | 6 microsatellites | Viability of young | Y |  |
| Milot et al 2013 | *Salmo salar* | Y | N | Y | 1 | 8 microsatellites | Fry assigned back to parent | Y |  |
| Bordeleau et al 2018 | *Salmo salar* | Y | N | N (tagging) | NA | NA | Survival to repeat spawning | Y |  |
| Jonsson et al 2019 | *Salmo salar* | Y | N | N (tagging) | NA | NA | Smolts per increasing proportion of wild females | Y |  |
| **O'Sullivan et al** | ***Salmo salar*** | **Y** | **Y** | **Y** | **6** | **30 microsatellites** | **Lifetime reproductive success** | **Y** |  |
| Morán et al 1991 | *Salmo trutta* | Y | Y | N (Detection of a specific allele) | NA | allozymes | Lack of introgression of *LDH-5*90* allele into wild population | Inferred |  |
| Hansen 2002 | *Salmo trutta* | Y | Y | N (population assignment) | NA | 9 microsatellites | Estimated admixture proportion | Y |  |
| Dannewitz et al 2004 | *Salmo trutta* | Y | N | Y | 1 | 11 microsatellites | Fry and parr assigned to parent | N |  |
| Reisenbichler & McIntyre 1977 | *Oncorhynchus mykiss* | Y | NA | N (marking/experimental design) | NA | allozymes | Survival, growth rate | Y |  |
| Chilcote et al 1986 | *Oncorhynchus mykiss* | Y | Y | N (Detection of a specific allele) | NA | allozymes | Proportional offspring survival | Y |  |
| Leider et al 1990 | *Oncorhynchus mykiss* | Y | Y | N (Detection of a specific allele) | NA | allozymes | Proportional offspring survival | Y |  |
| McLean et al 2004 | *Oncorhynchus mykiss* | Y | Y | N (population assignment) | NA | 8 microsatellites | Smolts per female | Y |  |
| Miller et al 2004 | *Oncorhynchus mykiss* | N | N | Y | 1 | 4-6 microsatellites | Offspring survival | Y |  |
| Araki et al 2007a, b, c | *Oncorhynchus mykiss* | Y | Y | Y | 3, 2 | 6 microsatellites | Lifetime reproductive success | Y |  |
| Araki et al 2009 | *Oncorhynchus mykiss* | Y | N | Y | 2 | 6 microsatellites | Lifetime reproductive success | Y |  |
| Ford et al 2006 | *Oncorhynchus kisutch* | Mixed | Mixed | Y | 1 | 6-7 microsatellites | Lifetime reproductive success | N |  |
| Thériault et al 2011 | *Oncorhynchus kisutch* | Y | N | Y | 2 | 10 microsatellites | Lifetime reproductive success | Y |  |
| Berejikian et al 2009 | *Oncorhynchus keta* | Y | N | Y | 1 | 8 microsatellites | Adult-to-fry survival | N |  |
| Hess et al 2012 | *Oncorhynchus tshawytscha* | Y | N | Y | 13 | 15 microsatellites | Lifetime reproductive success | N |  |
| Williamson et al 2010 | *Oncorhynchus tshawytscha* | Y | N | Y | 1 | 11 microsatellites | Estimated fitness based on fractional assignment | Y |  |

Table S2: Provenance- and sex-specific, and overall numbers of wild-bred and captive-bred, Atlantic salmon used for the RRS comparisons. For sex, F_Wild-bred_ = ‘Female wild-bred’, M_Captive-bred_ = ‘Male captive-bred’, etc.

| Provenance | | | Sex | | | |
| --- | --- | --- | --- | --- | --- | --- |
| Cohort | Wild-bred | Captive-bred | F_Wild-bred_ | F_Captive-bred_ | M_Wild-bred_ | M_Captive-bred_ |
| 1977 | 154 | 11 | 133 | 8 | 21 | 3 |
| 1978 | 77 | 64 | 69 | 39 | 8 | 25 |
| 1980 | 127 | 16 | 120 | 9 | 7 | 7 |
| 1981 | 155 | 100 | 133 | 63 | 22 | 37 |
| 1985 | 221 | 277 | 160 | 153 | 61 | 124 |
| 1989 | 334 | 328 | 195 | 209 | 139 | 119 |
| Overall | 1068 | 796 | 810 | 481 | 258 | 315 |

Table S3: Overall and sex-specific estimates of captive- and wild-bred Atlantic salmon mean absolute fitness used to estimate relative reproductive success, RRS, for each cohort. Numbers presented of offspring sampled, offspring assigned, parents sampled, and False Discovery Rate, FDR, were those used to correct RRS estimates as per^1^.

| Overall | | | | | | |
| --- | --- | --- | --- | --- | --- | --- |
| Cohort | Captive-bred fitness | Wild-bred fitness | Offspring sampled | Offspring assigned | Parents sampled | FDR |
| 1977 | 0.727 | 0.481 | 192 | 82 | 267 | 0.055 |
| 1978 | 0.344 | 1.180 | 213 | 113 | 172 | 0.055 |
| 1980 | 0.063 | 0.504 | 140 | 65 | 307 | 0.055 |
| 1981 | 0.170 | 0.974 | 296 | 168 | 293 | 0.055 |
| 1985 | 0.408 | 0.995 | 364 | 333 | 594 | 0.055 |
| 1989 | 0.223 | 0.365 | 242 | 195 | 696 | 0.055 |
|  |  |  |  |  |  |  |
|  |  |  |  |  |  |  |
| Female | | | | | | |
| Cohort | Captive-bred fitness | Wild-bred fitness | Offspring sampled | Offspring assigned | Parents sampled | FDR |
| 1977 | 0.125 | 0.534 | 192 | 72 | 267 | 0.036 |
| 1978 | 0.205 | 1.280 | 213 | 96 | 172 | 0.017 |
| 1980 | 0.111 | 0.517 | 140 | 63 | 307 | 0.030 |
| 1981 | 0.143 | 0.962 | 296 | 137 | 293 | 0.036 |
| 1985 | 0.399 | 1.031 | 364 | 226 | 594 | 0.058 |
| 1989 | 0.177 | 0.451 | 242 | 125 | 696 | 0.067 |
|  |  |  |  |  |  |  |
|  |  |  |  |  |  |  |
| Table S3 (cont.)  Male | | | | | | |
| Cohort | Captive-bred fitness | Wild-bred fitness | Offspring sampled | Offspring assigned | Parents sampled | FDR |
| 1977 | 2.333 | 0.143 | 192 | 10 | 267 | 0.046 |
| 1978 | 0.560 | 0.375 | 213 | 17 | 172 | 0.082 |
| 1980 | 0.000 | 0.286 | 140 | 2 | 307 | 0.297 |
| 1981 | 0.216 | 1.045 | 296 | 31 | 293 | 0.053 |
| 1985 | 0.419 | 0.902 | 364 | 107 | 594 | 0.140 |
| 1989 | 0.303 | 0.245 | 242 | 70 | 696 | 0.132 |

Table S4: Overall and cohort-specific variation in lifetime reproductive success (LRS) for wild-bred and captive-bred Atlantic salmon.

| Year of Spawning | Wild-bred | Captive-bred |
| --- | --- | --- |
| 1977 | 1.0356 | 2.0182 |
| 1978 | 2.9402 | 0.8958 |
| 1980 | 0.6964 | 0.0625 |
| 1981 | 1.6097 | 0.1829 |
| 1985 | 1.9500 | 0.6627 |
| 1989 | 0.8211 | 0.3387 |
| Overall | 1.4226 | 0.5022 |


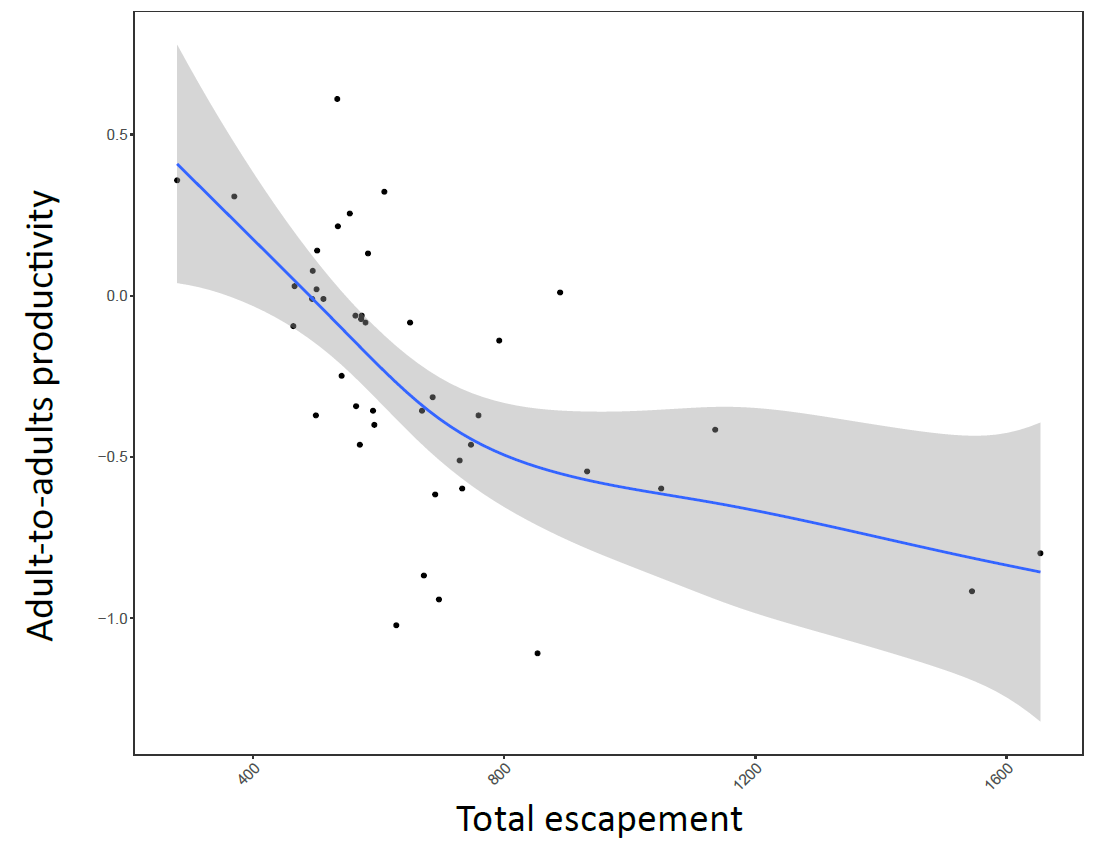


Figure S1: Stock-recruitment relationship between the total escapement (potential number of spawners) of wild- and captive-bred Atlantic salmon for a cohort and the natural logarithm of the productivity of that cohort, measured as recruits per spawner, giving an adult-to-adults productivity measure. The solid line represents the line-of-best fit from a GAM model, and shading represents the 95% confidence interval. Adjusted *R^2^* = 0.42, Deviance explained = 45.5%, *F_2.7_*= 9.11, *p*<0.001; note for *F*, reported degrees of freedom are estimated.


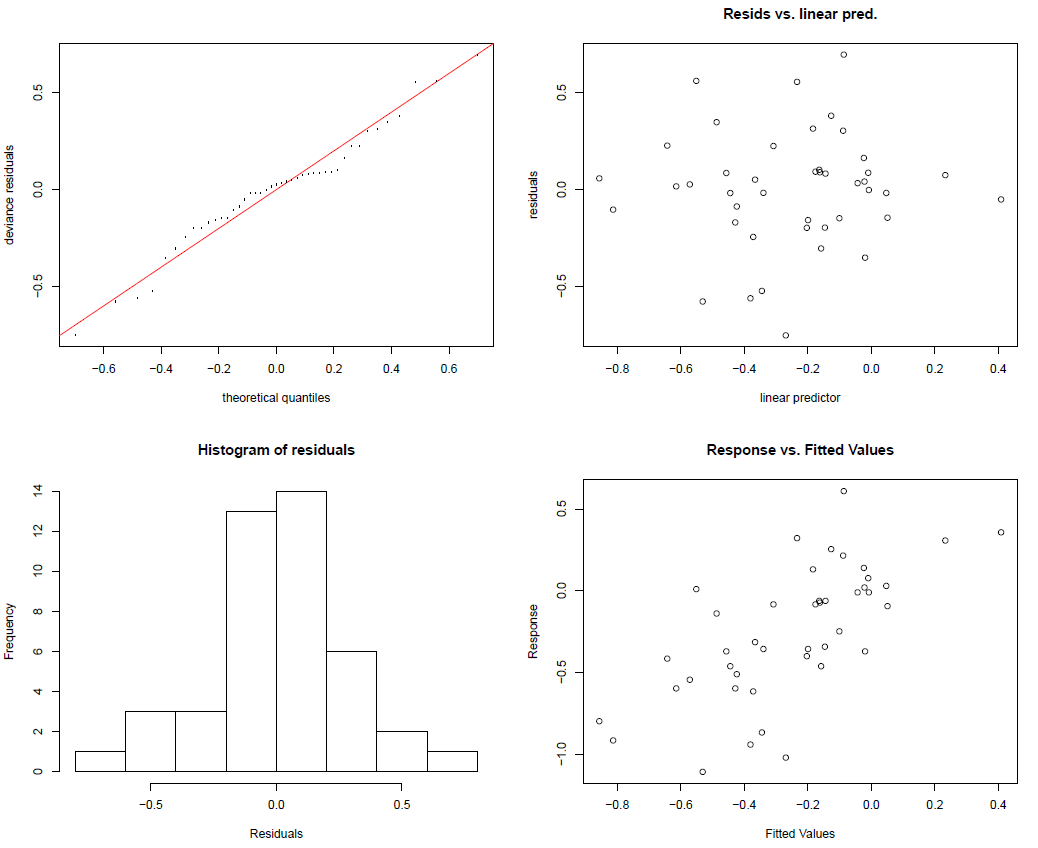


Figure 2: Diagnostic plots for stock-recruitment model.


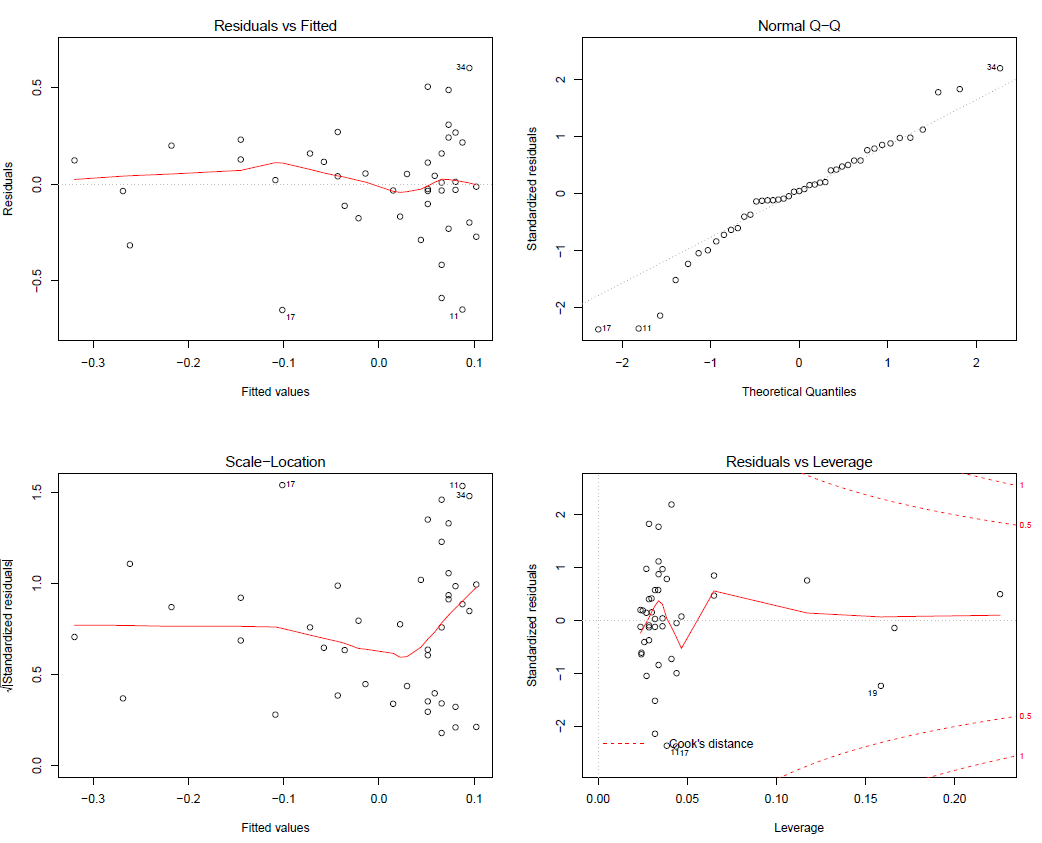


Figure S3: Diagnostic plots for linear model examining how larger proportions of captive-bred Atlantic salmon in the total number of fish that can potentially spawn reduces the productivity of the wild population.


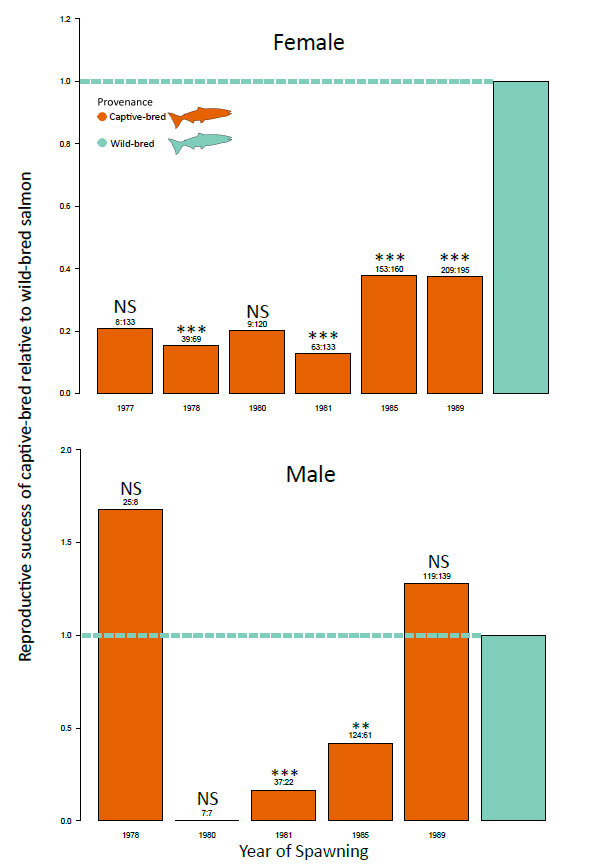


Figure S4: Cohort- and sex-specific comparisons of relative reproductive success, RRS, for captive-bred and wild-bred Atlantic salmon in the Burrishoole catchment, Ireland. Significance of cohort-specific comparisons determined using one-tailed permutation tests. Horizontal line for emphasis of increase/decrease in reproductive success of captive-born fish relative to wild-born fish. Numbers on top of bars represent the number of captive-bred (left number) salmon and wild-bred (right number) salmon used in cohort-specific comparisons. *: p <0.05, * *: p <0.01, ***: p <0.001. Note, to improve the figure’s readability, the male 1977 cohort is not displayed due to a very large (20.92) RRS estimate with large uncertainty bounds owing to a very low sample size for captive-bred males.


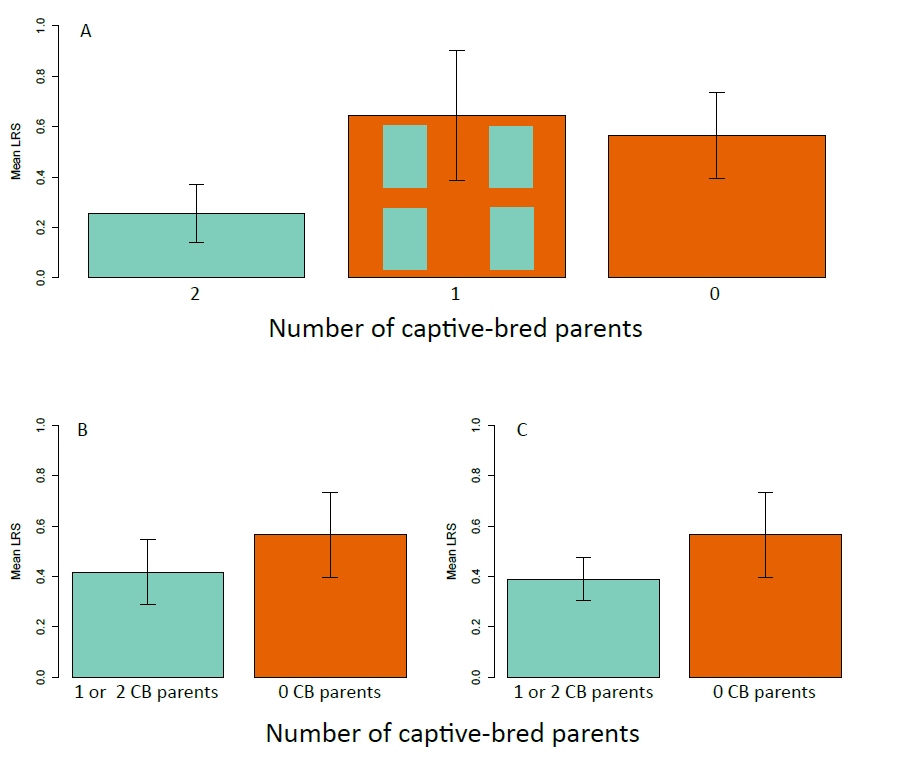


Figure S5: (A) Transgenerational effects of captive ancestry on the fitness of wild-bred Atlantic salmon in the Burrishoole catchment, Mayo, Ireland. Barplots represent the mean absolute fitness (measured as lifetime reproductive success, LRS) of wild-bred salmon for which both parents were known, separated into three categories: two captive-bred parents, one captive-bred parent (the other being wild-bred), no captive-bred parents (i.e. two wild-bred parents). One-tailed permutation tests revealed no significant difference in fitness between any of the categories (*p*-values; 1 captive-bred parent vs 0 captive-bred parents: 0.59, 2 captive-bred parents vs 0 captive-bred parents : 0.08, 2 captive-bred parents vs 1 captive-bred parent: 0.06). (B) Difference in mean LRS between wild-bred salmon with zero captive-bred parents versus those with one or two captive-bred parents. One-tailed permutation test revealed no significant difference in mean LRS between the two categories (*p* = 0.17). (C) Same as B but adding into the green category wild-bred salmon with one captive-bred parent and the other parent unknown. One-tailed permutation test revealed no significant difference in mean LRS between the two categories (*p* = 0.14). Error is represented by ±1 standard error.

Text S1: Bias caused by different sampling times for captive- and wild-bred fish

In the Burrishoole catchment, most captive-bred fish were sampled as upstream migrants (USM) on their return migration to spawn (94.74% USM, 5.26% DSM). Captive-bred fish were identified by an adipose fin clip that had been removed upon their release from the hatchery as ranched smolts. Similarly, most wild-bred fish were sampled as downstream migrants (DSM) post-spawning (94.21% DSM, 5.79% USM). If a captive-bred fish attempted to spawn, any resulting offspring would have to survive until they themselves were kelts for them to be sampled and, thus, assigned back to captive-bred parents (since the offspring would be wild-bred). That is, the offspring of wild-bred fish were sampled at the same life stage as their parents (both DSM), whereas the offspring of captive-bred parents were sampled at a later life stage than their parents (USM for parents, DSM for offspring). Therefore, any mortality that may have occurred in captive-bred adults (potential parents) between entry into fresh water (upstream migration) and subsequent re-entry into salt water as a kelt (downstream migration post-spawning) has the potential to bias downward the lifetime reproductive success, LRS, of captive-bred fish. To explore the extent of this bias, we performed a one-tailed permutation test comparing the non-zero reproductive success of captive- and wild-bred fish, that is, we removed records of fish that had zero LRS as these were potentially biasing downward the overall captive-bred LRS estimate. While many of these zeros are likely true zeros (that is, many captive-bred fish had zero LRS because they failed to spawn or none of their offspring survived to adulthood), we believed it pertinent to check whether removing such records changed the results in qualitative terms, which it did not. Therefore, this shows that captive-bred fish have lower fitness even after removing cases where LRS = 0, which removes this bias related to captive-bred fish and wild-bred fish being sampled at different times, and also discounts the fact that a higher fraction of captive-bred fish may have simply failed to spawn (and hence left no offspring). This gives us confidence that our overall result – that captive-bred fish have lower fitness in the wild than wild-bred fish – is not entirely driven by a potential bias due to differential sampling times. However, removing the LRS = 0 cases also makes for a more conservative test of fitness differences, given that many of these were likely true zeros. We do, however, acknowledge that the RRS estimate based on including the zeros may be an overestimate. We arrived at the same qualitative result whether using individual-level (pedigree-derived LRS) data or population-level (productivity) data: namely, that the expected mean fitness of the mixed population is lower than that of a hypothetically “pure” population. The population-level data indicated a percentage reduction here of ~22%, while the individual-level data indicated a reduction of ~10%, with the discrepancy likely related to differences in time-series length (43 cohorts for the population-level analysis; 6 cohorts for the individual-level analysis), incomplete sampling for the individual-level analysis, and the fact that the population-level analysis involved correcting for density dependence.

Text S2: Ova-per-ovum productivity.

We ‘converted’ adults into eggs by summing the estimated ova deposition by wild-bred grilse recruits in year *t*+4 and wild-bred MSW recruits in year *t*+5, and dividing by the estimated ova deposition of wild-bred and captive-bred spawners in year *t*. This ‘ova-per-ovum’ measure of productivity was strongly correlated (Pearson’s correlation: *t* = 22.63, *df* = 41, *r* = 0.96, *p*< 0.001) with our recruits per spawner productivity measure.

The productivity of a given Atlantic salmon cohort can be estimated as the number of adult recruits divided by the number of potential breeders in year *t* that produced those recruits. Since wild-bred Atlantic salmon in our system typically exhibit either a ‘2+,grilse’ or ‘2+,1MSW’ lifecycle, we assume this population structure for all analyses. However, this difference in sea age means that not all offspring originating from a given spawning cohort will themselves recruit into the same future spawning cohort. Therefore, the total wild-bred productivity for a given spawning cohort could be estimated as

(Eq.1) *P_Wild-bredt_* = (Ʃ*Ngrilse_t+4_* + Ʃ*Nmsw*_t+5_)/*Nspawners_t_*

where *P_Wild-bredt_* is the productivity of a wild-bred cohort at *t*, *Ngrilse_t+4_* is the number of salmon recruiting at *t+4*, *Nmsw_t+5_* is the number of salmon recruiting at *t+5*, and *Nspawners_t_* is the total number of salmon (both captive-bred and wild-bred) spawning at *t*. However, MSW salmon and grilse do not contribute equally to productivity due to female MSW salmon having greater fecundity than female grilse^24^. Therefore, using the fecundity of grilse and MSW salmon at *t+4* and *t+5*, respectively should provide a more accurate estimate as it effectively examines the number of zygotes produced by a given cohort.

Atlantic salmon fecundity can be estimated from either length or weight data, using the well-documented relationship between fish size and fecundity^24^. In Burrishoole, lengths of grilse (either as upstream migrating spawners or downstream migrating kelts) are documented every year, with fecundity estimated using the nonlinear function

(Eq.2) *F_i_ = e*^(^*^5.79+0.035 length^_i_*^)^

where *F* and *length* are the estimated fecundity and measured length for the *ith* female, respectively (Marine Institute, unpublished data). A sex ratio of 55% female : 45% male is used. The fecundity of MSW fish was calculated using a standard value of 4274 ova per female, with a sex ratio of 70% female : 30% male. The fecundity of captive bred fish was calculated using an average number of ova per female, based on actual ova counts in the broodstock, and a sex ratio of 55% female : 45% male.

Equation 1 can now be alternatively parameterized as

(Eq.3) *P_Wild-bredt_* = (Ʃ*Fgrilse_t+4_* + Ʃ*Fmsw_t+5_*)/*Fspawners_t_*

where *Fgrilse_t+4_* and *Fmsw_t+5_* are the estimated fecundities of individual grilse and MSW salmon at *t+4* and *t+5*, respectively, and *Fspawners_t_* is the total fecundity of all spawning females (both captive-bred and wild-bred) at *t*. *P_Wild-bredt_* is now a measure of ova-per-ovum productivity that is robust to both variation in lifecycle and fecundity. We estimated productivity for all years where data were available. Due to the stocking of captive-bred ova within the catchment for experimental purposes in some years, *Fspawners_t_* was corrected as necessary (Marine Institute, pers. comm.). We repeated the population level analyses using ova per ovum productivity instead of recruits per spawner. The results were qualitatively unchanged.

Text S3: Results of the test for a higher proportion of zero LRS for captive-bred than wild-bred Atlantic salmon.

The proportion of captive-bred and wild-bred fish that displayed zero LRS was 0.82 and 0.67, respectively. The 95% confidence interval for these estimates was 0.12 – 1.0. *Χ^2^* = 72.45, *df* = 1, *p* < 0.001

**Supplementary References**

1. Araki H, Blouin MS. Unbiased estimation of relative reproductive success of different groups: evaluation and correction of bias caused by parentage assignment errors. 2005. Mol. Ecol. 14:4097-4109. doi:10.1111/j.1365-294X.2005.02689.x
2. Araki H, Waples RS, Ardren WR, Cooper B, Blouin MS. 2007. Effective population size of steelhead trout: influence of variance in reproductive success, hatchery programs, and genetic compensation between life-history forms. Mol. Ecol. 2007.16(5):953–66. doi:10.1111/j.1365-294X.2006.03206.x
3. Araki H, Cooper B, Blouin MS. Genetic Effects of Captive Breeding Cause a Rapid, Cumulative Fitness Decline in the Wild. 2007. Science 318(5847):100–3. doi: 10.1126/science.1145621
4. Araki H, Ardren WR, Olsen E, Cooper B, Blouin MS. Reproductive success of captive-bred steelhead trout in the wild: evaluation of three hatchery programs in the Hood River.2007. Conserv. Biol. 21(1):181-190. doi:10.1111/j.1523-1739.2006.00564.x
5. Araki H, Cooper B, Blouin MS. Carry-over effect of captive breeding reduces reproductive fitness of wild-born descendants in the wild. 2009. Biol. Lett. 5(5):621–4. doi:10.1098/rsbl.2009.0315
6. Berejikian BA, Van Doornik DM, Scheurer JA, Bush R. Reproductive behavior and relative reproductive success of natural- and hatchery-origin Hood Canal summer chum salmon (Oncorhynchus keta). 2009. Can. J. Fish Aquat. Sci. 66(5):781-789. doi:10.1139/F09-041
7. Bordeleau X, Hatcher BG, Denny S, Fast MD, Whoriskey FG, Patterson DA, Crossin GT. Consequences of captive breeding: Fitness implications for wild-origin, hatchery-spawned Atlantic salmon kelts upon their return to the wild. 2018. Biol. Conserv. 225:144–53. doi: 10.1016/j.biocon.2018.06.033
8. Chilcote MW, Leider SA, Loch JJ. Differential Reproductive Success of Hatchery and Wild Summer-Run Steelhead under Natural Conditions. 1986. Trans. Am. Fish Soc. 115(5):726–35. doi: 10.1577/1548-8659(1986)115<726:DRSOHA>2.0.CO;2
9. Dannewitz J, Petersson E, Dahl J, Prestegaard T, Löf A-C, Järvi T. Reproductive success of hatchery‐produced and wild‐born brown trout in an experimental stream. 2004. J. Appl. Ecol. 41(2):355-364. doi:10.1111/j.0021-8901.2004.00895.x
10. Fleming IA, Lamberg A, Jonsson B. Effects of early experience on the reproductive performance of Atlantic salmon. 1997. Behav. Ecol. 8(5):470–80. doi: doi.org/10.1093/beheco/8.5.470
11. Ford MJ, Fuss H, Boelts B, LaHood E, Hard J, Miller J. Changes in run timing and natural smolt production in a naturally spawning coho salmon (*Oncorhynchus* *kisutch*) population after 60 years of intensive hatchery supplementation. 2006. Can. J. Fish Aquat. Sci. 63(10):2343-2355. doi:10.1139/f06-119
12. Hansen MM. Estimating the long-term effects of stocking domesticated trout into wild brown trout (Salmo trutta) populations: an approach using microsatellite DNA analysis of historical and contemporary samples. Mol. Ecol. 2002. 11(6):1003‐1015. doi:10.1046/j.1365-294x.2002.01495.x
13. Hess MA, Rabe CD, Vogel JL, Stephenson JJ, Nelson DD, Narum SR. Supportive breeding boosts natural population abundance with minimal negative impacts on fitness of a wild population of Chinook salmon. 2012. Mol. Ecol. 21(21):5236-5250. doi:10.1111/mec.12046
14. Jonsson B, Jonsson N, Jonsson M. Supportive breeders of Atlantic salmon *Salmo salar* have reduced fitness in nature. 2019. Conserv. Sci. Pract. 1(9); e85. doi: 10.1111/csp2.85
15. Leider SA, Hulett PL, Loch JJ, Chilcote MW. Electrophoretic comparison of the reproductive success of naturally spawning transplanted and wild steelhead trout through the returning adult stage. 1990. Aquaculture. 88(3–4):239–52. doi: 10.1016/0044-8486(90)90151-C
16. McGinnity P, Prodöhl P, Ferguson A, Hynes R, Ó Maoiléidigh N, Baker N, Cotter D, O’Hea B, Cooke D, Rogan G, Taggart J, Cross T. Fitness reduction and potential extinction of wild populations of Atlantic salmon, Salmo salar, as a result of interactions with escaped farm salmon. 2003. Proc. R. Soc. Lond. B Biol. Sci. 270(1532):2443–50. doi:10.1098/rspb.2003.2520
17. McLean JE, Bentzen P, Quinn TP. Differential reproductive success of sympatric, naturally spawning hatchery and wild steelhead, *Oncorhynchus* *mykiss*. (2004). Environ. Biol. Fishes 69:359-369. doi: 10.1023/B:EBFI.0000022875.78560.ce
18. Miller LM, Close T, Kapuscinski AR. Lower fitness of hatchery and hybrid rainbow trout compared to naturalized populations in Lake Superior tributaries. 2004. Mol. Ecol. 13(11):3379–88. doi: 10.1111/j.1365-294X.2004.02347.x
19. Milot E, Perrier C, Papillon L, Dodson JJ, Bernatchez L. Reduced fitness of Atlantic salmon released in the wild after one generation of captive breeding. 2012. Evol. Appl. 2013. 6(3):472-485. doi:10.1111/eva.12028
20. Morán P, Pendás AM, Garcia-Vázquez E, Izquierdo J. Failure of a stocking policy, of hatchery reared brown trout, *Salmo trutta* L., in Asturias, Spain, detected using *LDH-5* * as a genetic marker. 1991.J Fish Biol. 39:117–21. doi: 10.1111/j.1095-8649.1991.tb05075.x
21. Reisenbichler RR, McIntyre JD. Genetic Differences in Growth and Survival of Juvenile Hatchery and Wild Steelhead Trout, *Salmo* *gairdneri*. 1977. Journal of the Fisheries Research Board of Canada. 34(1):123-128. doi:[10.1139/f77-015](https://doi.org/10.1139/f77-015)
22. Thériault V, Moyer GR, Jackson LS, Blouin MS, Banks MA. Reduced reproductive success of hatchery coho salmon in the wild: insights into most likely mechanisms. 2011. Mol. Ecol. 20(9):1860–9. doi:10.1111/j.1365-294X.2011.05058.x
23. Williamson KS, Murdoch AR, Pearsons TN, Ward EJ, Ford MJ. Factors influencing the relative fitness of hatchery and wild spring Chinook salmon (Oncorhynchus tshawytscha) in the Wenatchee River, Washington, USA. 2010. Can. J. Fish Aquat. Sci. 67(11):1840-1851. doi:10.1139/F10-099
24. de Eyto E, White J, Boylan P, Clarke B, Cotter D, Doherty D, Gargan P, Kennedy R, McGinnity P, O’Maoiléidigh N. The fecundity of wild Irish Atlantic salmon Salmo salar L. and its application for stock assessment purposes. 2015. Fish. Res. 164: 159–169. doi:10.1016/j.fishres.2014.11.017
